# Structure Elucidation and Anticancer Activity of 7-Oxostaurosporine Derivatives from the Brazilian Endemic Tunicate *Eudistoma vannamei*

**DOI:** 10.3390/md10051092

**Published:** 2012-05-21

**Authors:** Paula Christine Jimenez, Diego Veras Wilke, Elthon Gois Ferreira, Renata Takeara, Manoel Odorico de Moraes, Edilberto Rocha da Silveira, Tito Monteiro da Cruz Lotufo, Norberto Peporine Lopes, Leticia Veras Costa-Lotufo

**Affiliations:** 1 Institute of Marine Sciences, LABOMAR, Federal University of Ceará, Fortaleza, CE 60165-081, Brazil; Email: paulacjimenez@gmail.com (P.C.J.); elthonferreira@gmail.com (E.G.F.); tmlotufo@ufc.br (T.M.C.L.); 2 Department of Physiology and Pharmacology, School of Medicine, Federal University of Ceará, Fortaleza, CE 60430-270, Brazil; Email: diegowilke@gmail.com (D.V.W.); odorico@ufc.br (M.O.M.); 3 Department of Chemistry and Physics, Faculty of Pharmaceutical Sciences of Ribeirão Preto, University of São Paulo, Ribeirão Preto, SP 14040-903, Brazil; Email: rtakeara@yahoo.com (R.T.); npelopes@fcfrp.usp.br (N.P.L.); 4 Department of Organic and Inorganic Chemistry, Federal University of Ceará, Fortaleza, CE 60021-970, Brazil; Email: edil@ufc.br

**Keywords:** cell cycle arrest, ascidians, staurosporine

## Abstract

The present study reports the identification of two new staurosporine derivatives, 2-hydroxy-7-oxostaurosporine (**1**) and 3-hydroxy-7-oxostaurosporine (**2**), obtained from mid-polar fractions of an aqueous methanol extract of the tunicate *Eudistoma vannamei*, endemic to the northeast coast of Brazil. The mixture of **1** and **2** displayed IC_50_ values in the nM range and was up to 14 times more cytotoxic than staurosporine across a panel of tumor cell lines, as evaluated using the MTT assay.

## 1. Introduction

Tunicates are a group of marine invertebrates particularly abundant along coastal regions, often found as the dominant organisms in sessile benthic communities [1]. In Brazil, as in many other regions, information regarding these animals is restricted to faunal inventories and, in many cases, is inaccurate or incomplete [2]. In regards to Brazilian tropical waters, the deficiency in species inventories is quite evident, as very few references are available [3,4,5].

**Figure 1:**
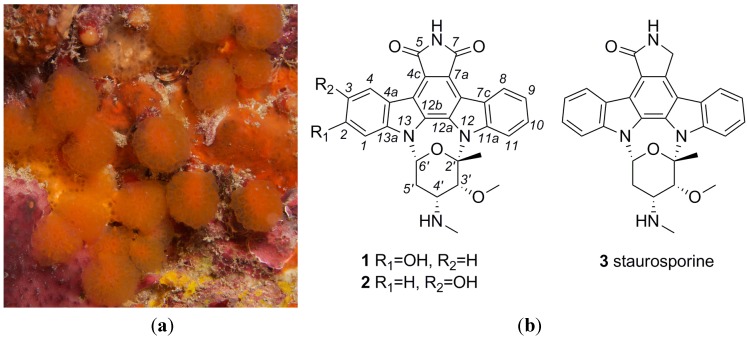
(**a**) *Eudistoma vannamei*; (**b**) Structures of 2-hydroxy-7-oxostaurosporine (**1**), 3-hydroxy-7-oxostaurosporine (**2**) and staurosporine (**3**).

The faunal list for the state of Ceará, on the northeastern coast of Brazil, indicated that 22 species of ascidians were registered [5,6], including novel species and many cases of regional endemism. This coastline has been scarcely explored as a source of natural products. In 2003, a pioneering study investigated the cytotoxicity of aqueous methanol extracts of the ten most abundant ascidians of Ceará. The results of these assays suggested that *Eudistoma vannamei* Millar, 1977 (Figure 1 a) might be of interest due to its marked bioactivity, especially the inhibition of growth in tumor cell lines [7]. Evaluation of the cytotoxicity data from these extracts suggested the presence of amino acid-derived compounds in fractions of intermediate polarity. Treatment of leukemia cells with the amino acid-derived compounds resulted in apoptosis [8].

The genus *Eudistoma* is the most diverse among the Polycitoridae, with most of its species living in tropical regions [9]. On the Brazilian coast, only one species of *Eudistoma* was found in temperate waters [10], while in the warmer Northeast region, there are at least seven species of this genus. *E. vannamei* is endemic to the Northeast coast of Brazil where it forms large colonies on the underside of ledges of the beach rocks and is characterized by its conspicuous orange or yellow bulbus heads [3,5]. *E. vannamei*’s sessile habit and apparent lack of mechanical defenses would make it an easy target for predators. However, taking into account its dominance on the intertidal reefs, the species most probably relies on chemical deterrents for protection.

Several cytotoxic substances have been isolated from *Eudistoma* ascidians, including the alkaloids eudistomins A–T, eudistomidins A–F and eudistalbin [11,12,13,14,15,16,17,18,19] and the macrolides iejimalides A–D [20,21]. The present study reports the bioactivity-guided isolation of two new members of the staurosporine group of alkaloids from the extract of *E. vannamei*.

## 2. Results and Discussion

The aqueous methanol extract from *E. vannamei* was submitted to cytotoxicity-guided fractionation (flowchart provided in the supplementary material), resulting in the isolation of 5 mg of an active fraction, which appeared as a single spot by TLC and a single peak via LC-MS analysis.

This material was submitted to NMR and HRMS analysis. The ESI-HRMS spectrum showed one signal at *m/z* 497.1830 (error value: 2.21 ppm), suggesting the molecular formula C_28_H_24_N_4_O_5_. The initial analysis of the ^13^C NMR contained more carbons than expected, and appeared as two sets of peaks with similar chemical shifts. Detailed analysis of the MS and MS/MS data along with evaluation of the ^13^C NMR data suggested that the material was a mixture of two staurosporine derivatives, compounds **1** and **2**. 

**Figure 2:**
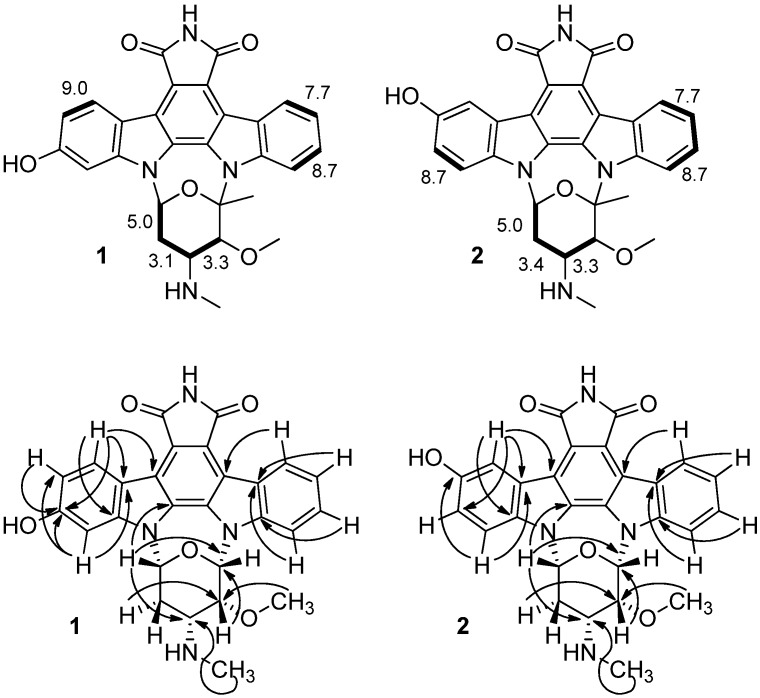
A summary of the NMR data including (**top**) coupling constants and COSY correlations (**bottom**) key long-range ^1^H,^13^C-correlations (depicted by arrows) observed in the HMBC experiment.

A detailed set of NMR spectral data was then collected including ^1^H, ^13^C, COSY, HSQC and HMBC spectra to identify the structures of **1** and **2**. Each of the protons in **1** and **2** were assigned using a combination of chemical shift data and coupling constants from the ^1^H NMR spectrum (Table 1) and correlations from a COSY spectrum (Figure 2). Analysis of the chemical shift and coupling constant data indicated that the glycosidic unit matched that in staurosporine [22,23,24,25]. An HSQC experiment enabled the assignments of each methine, methylene and methyl carbon (Table 1). The COSY spectrum revealed the scalar coupling correlations for the most deshielded proton attached to sp^3^ carbons H-6′ (δ_H_ 6.62 for **1** and 6.69 for **2**) with the heterotopic methylene protons 2H-5′ (δ_H_ 2.36/2.72 for **1** and δ_H_ 2.46/2.74 for **2**) which, in turn, showed geminal coupling. The oxymethine protons at δ_H_ 3.95 (H-3′ for **1**) and 3.97 (H-3′ for **2**) showed correlations with the azomethine protons at δ_H_ 3.23 and 3.26 (H-4′) for **1** and **2**, respectively. In the aromatic region, the most deshielded proton at δ_H_ 9.94 (H-8 for both **1** and **2**) coupled to one at δ_H_ 7.46 (H-9 for both **1** and **2**), which coupled with one at δH 7.61 (H-10 for both **1** and **2**) and that, in turn, coupled with the ones at δ_H_ 8.15 (for **1**) and 8.17 (for **2**). On the hydroxyl substituted benzene ring, the most deshielded doublet (*J* = 8.4 Hz) at δ_H_ 9.63 (H-4 for **1**) correlated with the one at δ_H_ 7.40 (H-3 of **1**), while the correspondent H-4 of **2** at δ_H_ 9.58 (br, d) coupled weakly with the proton at δ_H_ 7.61 that, in turn, coupled with the one at δ_H_ 7.54 (d, *J* = 8.7 Hz, H-1 of **2**). 

**Table 1:** NMR Data (500 MHz, pyridine-*d*_5_) for 2-hydroxy-7-oxostaurosporine (**1**) and 3-hydroxy-7-oxostaurosporine (**2**).

	2-Hydroxy-7-oxostaurosporine (1)			3-Hydroxy-7-oxostaurosporine (2)		
Position	δ_C_, mult.	δ_H_ ( *J* in Hz)	HMBC ^d^	δ_C_, mult.	δ_H_ ( *J* in Hz)	HMBC ^d^
1	95.6, CH	7.39 br. s	2, 3, 4a	109.7, CH	7.54 d (8.7)	3
2	159.5, C			117.4, CH	7.61 ^c^	3
3	111.3, CH	7.40 d (9.6)	4a	153.8, C		
4	126.6, CH	9.63 d (8.4)	2, 4b, 13a	111.7, CH	9.58 br. d	2, 13a
4a	115.8, C			123.8, C		
4b	117.0, C			117.0, C		
4c	120.2 ^a^, C			120.0 ^a^, C		
5	172.7 ^b^, C			172.6 ^b^, C		
7a	123.4 ^a^, C			122.1 ^a^, C		
7b	116.8, C			116.8, C		
7c	124.7, C			124.7, C		
8	125.8, CH	9.94 d (7.9)	7b, 10, 11a	125.9, CH	9.94 d (7.9)	7b, 10, 11a
9	120.7, CH	7.46 d (7.5)	7c, 11	120.7, CH	7.46 t (7.5)	7c, 11
10	126.4, CH	7.61 ^c^	8, 11a	126.4, CH	7.61^c^	8, 11a
11	116.1, CH	8.15 d (8.7)	7c, 9	116.8, CH	8.17 d (8.7)	7c, 9
11a	141.9, C			141.8, C		
12a	132.3, C			132.4, C		
12b	131.2, C			131.8, C		
13a	141.1, C			133.3, C		
2′	91.7, C			91.8, C		
3′	84.1, CH	3.95 d (3.3)	2′, CH_3_O, CH_3_	84.2, CH	3.97 d (3.3)	2′, CH_3_O, CH_3_
4′	50.8, CH	3.23 br. q (3.1)	2′, 3′, 6′, CH_3_N	50.8, CH	3.26 br. q (3.4)	2′, 3′, 6′, CH_3_N
5′	29.9, CH_2_	2.72 m	3′, 4′, 6′	29.8, CH_2_	2.74 m	3′, 4′, 6′
		2.36 m			2.46 m	
6′	80.8, CH	6.62 d (5.0)	2′, 4′, 12b	80.9, CH	6.69 d (5.0)	2′, 4′, 12b
CH_3_-NH	30.5, CH_3_	1.48 s	4′	30.5, CH_3_	1.48 s	4′
CH_3_O-C_3′_	57.2, CH_3_	3.31 s	3′	57.2, CH_3_	3.32 s	3′
CH_3_-C_2′_	33.8, CH_3_	2.37 s	2′, 3′	33.8, CH_3_	2.38 s	2′, 3′

^a,b^ Values with the same superscript are interchangeable in the same column; ^c^ Due to partial overlapping the multiplicity and *J* values could not be determined precisely; ^d^ HMBC correlations, optimized for 7.25 Hz, are from proton(s) stated to the indicated carbon.

Subsequent HMBC data facilitated the assignment of the remaining carbons (Table 1) with the exception of C-4c, C-5, C-7 and C-7a, which were interpreted by comparison with the literature [22,23,24,25]. Further long range correlations observed in the HMBC spectrum (Figure 2) provided definitive proof for the connectivity and thereby confirmed the structural assignment of **1** and **2** as 2-hydroxy-7-oxostaurosporine and 3-hydroxy-7-oxostaurosporine, respectively.

Staurosporines are a group of highly cytotoxic indole-carbazole alkaloids of which the parental molecule was isolated in 1977 from the fermentation broth of the soil actinomycete *Streptomyces staurosporeus* in a screening protocol directed to identify PKC (protein kinase C) inhibitors [26]. Currently, the staurosporines are a group of over 50 structurally related substances of natural and synthetic origins and have been found frequently in extracts from *Eudistoma* tunicates. 11-Hydroxystaurosporine and 3,11-dihydroxystaurosporine were isolated from *Eudistoma* sp. collected on a Micronesian island [27], while the staurosporine aglicone (K252-c) was obtained from another *Eudistoma* species found on the west coast of Africa [28]. Between 1999 and 2002, Schupp and co-workers published three articles covering the isolation and nM-range cytotoxicity of five new (3-hydroxy-4′-*N*-methylstaurosporine, 3-hydroxy-4′-*N*-demethylstaurosporine, 3′-demethoxy-3′-hydroxy-4′-*N*-demethylestaurosporine, 3-hydroxy-3′-demethoxy-3′-hydroxystaurosporine and 11-hydroxy-4′-*N*-demethylstaurosporine) and seven previously known staurosporines found in the Micronesian ascidian *Eudistoma toealensis* and its predatory flatworm *Pseudoceros* sp. [24,29,30].

The mixture of compounds **1**–**2** presented strong cytotoxic activity against a panel of seven human tumor cell lines (HL-60, Molt-4, Jurkat, K562, HCT-8, SF-295 and MDA-MB-435) and normal proliferating lymphocytes (PBMC), with an IC_50_ ranging from 10.33 nM in Jurkat leukemia cells to 687.08 nM in normal PBMC cells (Table 2). Standard staurosporine (**3**), used as a positive control, was generally less potent than compounds **1**–**2** against tumor cells. Against normal proliferating lymphocytes, both **3** and **1**–**2** presented similar activity, suggesting a better selectivity index for **1**–**2** (Table 2). For example, **1**–**2** was 26.46 times more active against HL-60 cells than against PBMC, whereas STP was 2.00 times more active against HL-60 cells when compared with normal cells. 

**Table 2:** Cytotoxicity of 2-hydroxy-7-oxostaurosporine/3-hydroxy-7-oxostaurosporine (**1**/**2**) and staurosporine (**3**), evaluated using the MTT assay after 72 h of incubation. The IC_50_ (nM) values and 95% CI were obtained via nonlinear regression (first values); the 95% CI are set in brackets.

Cell Line	IC_50_ (nM) 1–2	Selectivity Index ^a^ PBMC *vs*. Cancer Cells	IC_50_ (nM) 3	Selectivity Index ^a^ PBMC *vs*. Cancer Cells
HL-60	25.97 [22.42–30.09]	26.46	391.83 [316.81–484.86]	2.00
Molt-4	18.64 [15.97–21.74]	36.86	154.50 [128.12–186.33]	5.08
Jurkat	10.33 [7.12–15.00]	70.08	83.96 [51.38–137.25]	9.34
K562	144.47 [103.88–200.9]	4.75	1960.86 N.C.	0.40
HCT-8	58.24 [50.96–66.58]	11.80	83.83 [66.43–105.80]	9.36
SF 295	57.90 [47.10–71.16]	11.87	569.52 [444.13–730.28]	1.38
MDA MB 435	28.68 [25.64–32.06]	23.96	215.42 [153.64–301.80]	3.64
PBMC	687.08 [452.55–1043.48]	-	784.51 [566.95–1085.89]	

N.C.: value was not converted; ^a^ ratio between the cytotoxicities expressed as IC_50_ (nM) against peripheral blood mononucleated cells (PBMCs) (normal cells) and cancer cell lines.

Structure-activity relationship studies involving staurosporine and structurally related compounds have shown that slight changes in structure lead to drastic changes in functionality or, essentially, selectivity towards a target [31,32,33,34]. A preliminary investigation on the mode of action of compounds **1** and **2** was conducted using HL-60 leukemia cells as a model. The mixture of **1** and **2** (80 nM) induced a notable and sustained cytostatic effect against HL-60 cells throughout the 72 h analysis. This observation was accompanied by an increase in the accumulation of cells in G2/M and a decrease of those in G0/G1 and S phases. The toxicity of STP (430 nM), however, was kinetically less consistent. Moreover, after 24 h, almost the entire STP-treated culture was arrested in G2/M, which was followed by an increase in polyploid cells at further time points, showing a less strict blockage. When the concentration of the mixture was increased to 160 nM, extensive DNA damage occurred, leading to cellular apoptosis (results not shown). However, in order to access the contribution of each isomer in the observed antiproliferative activity, it will be necessary to isolate the compounds and to access the mode of action of each one individually. 

## 3. Experimental Section

### 3.1. Reagents

Cell culture media, fetal bovine serum and antibiotics were acquired from Gibco (Grand Island, NY, USA). Staurosporine, MTT and resazurin were obtained from Sigma-Aldrich (St. Louis, MO, USA). All other reagents used were of analytical grade.

### 3.2. Collection and Identification of *Eudistoma vannamei*

Samples of *Eudistoma vannamei* were collected in crevices or on the underside of beach rocks in the intertidal zone of Taíba Beach (03°34,931′S; 038°54,469′W), on the west coast of Ceará state, Brazil. The material was immediately immersed in methanol and stored at −4 °C. Part of the material was fixed in 70% ethanol and sent for identification. A voucher specimen #198 has been deposited at the ascidian collection from the Institute of Marine Sciences, Federal University of Ceará (Instituto de Ciências do Mar, Universidade Federal do Ceará).

### 3.3. Extraction and Bioguided Fractionation

The collected specimens (8.8 kg) were extracted with MeOH (1:5, m/v, wet weight). The suspension was filtered, concentrated under reduced pressure (TECNAL, model TE-120) and lyophilized (Thermo Electron Corporation, model: MicroModulyo Freeze Dryer 115) to obtain the dry raw methanol extract (351.80 g). The raw extract was then resuspended in MeOH to precipitate the salt, filtered and afterwards diluted with water to a proportion of 7:3 (MeOH:H_2_O). A successive partition of the aqueous phase followed, first with CH_2_Cl_2_ (2:1, v/v, 3-fold) and then with *n*-BuOH (2:1, v/v, 3-fold). The solvent of all the fractions was removed under reduced pressure. The CH_2_Cl_2_ partition was fractionated by flash chromatography on a glass column (55 × 25 cm) filled with 800 g of silica gel 60 GF_254_ 70–230 mesh ASTM (Sigma), using an *n*-hexane/EtOAc gradient from 20–100% EtOAc. After analysis with thin layer chromatography (TLC), 20 fractions (1 to 20) were obtained and further studied. Fraction 14 (1.81 g) exhibited the most promising biological potential and therefore was subjected to flash chromatography on silica gel 60 GF_254_ (glass column 55 × 25 cm; 170 g silica) in an isocratic elution with CH_2_Cl_2_:MeOH (9:1, v/v), which provided six sub-fractions. Further purification, after the biological evaluation, led to the selection of sub-fraction 3 (*i.e*., SF3), which was purified by TLC. The major sub-sub-fraction 1 (*i.e*., SF3.1) after sequential TLC resulted in one active mixture of isomers (5 mg). The structure elucidation was based on infrared spectra (IR; Perkin-Elmer, model FT-IR 1000), nuclear magnetic resonance (NMR) and high-resolution mass spectrometry. All NMR experiments were recorded on a Bruker DRX-500 spectrometer operating at 499.80 and 125.69 MHz for ^1^H and ^13^C, respectively, using standard pulse sequences supplied by the manufacturer. The ESI-MS was performed with Bruker Daltonics™ equipment (UltrO-TOF, Billerica, MA, USA). The sample (0.5 µg/mL) was dissolved in methanol/water at a 1:1 ratio and was introduced into the electrospray source at 5 µL/min via an infusion pump (Cole-Parmer, Vernon Hills, IL, USA). Nitrogen was used as a nebulising gas, and the capillary voltage was set to 3500 V.

### 3.4. Evaluation of Cytotoxicity

#### 3.4.1. Cell Lines and Cell Models

HL-60 (promyelocytic leukemia), Molt-4 (lymphocytic leukemia), Jurkat (T cell leukemia), K562 (chronic myeloid leukemia) HCT-8 (colon cancer), MDA MB-435 (melanoma), and SF-295 (glioblastoma) human tumor cell lines were obtained from the National Cancer Institute, Bethesda, MD, USA. Cells were grown in RPMI-1640 medium supplemented with 10% fetal bovine serum, 2 mM glutamine, 100 μg/mL streptomycin and 100 U/mL penicillin and incubated at 37 °C under a 5% CO_2_ atmosphere. 

Peripheral blood mononucleated cells (PBMCs) were used as a model for the evaluation of cytotoxicity in normal cells. Peripheral blood samples were obtained from four healthy volunteers. Venous blood (8 mL) was collected via routine venipuncture into a sterile tube with EDTA and carefully layered over 2 mL of Ficoll-Histopaque (Sigma-Aldrich, St. Louis, MO, USA) and centrifuged at 1500 rpm for 20 min. The layer containing the lymphocytes was aspirated, washed twice with PBS and tested for viability using trypan blue. PBMCs were suspended to their final concentration in RPMI medium supplemented with 20% fetal bovine serum, 2 mm glutamine, 100 μg/mL streptomycin, 100 U/mL penicillin and 3% phytohemagglutinin to stimulate proliferation.

#### 3.4.2. MTT Assay

Cells were plated into 96-well plates (3 × 10^5^ cells/mL for suspended leukemia cells and 1 × 10^5^ cells/mL for adherent solid tumor cells). Adherent cells were plated 24 h prior to addition of test substances, which were added using the HTS, and incubated for 72 h. Control groups received DMSO. Three hours before the end of the incubation, 150 µL of a stock solution (0.5 mg/mL) of MTT (3-(4,5-dimethyl-2-thiazolyl)-2,5-diphenyl-2*H*-tetrazolium bromide; Sigma-Aldrich Co., St. Louis, MO, USA) was added to each well. Absorbance was measured using a multiplate reader (DTX 880 Multimode Detector, Beckman Coulter, Inc. Fullerton, CA, USA). The effect was quantified as the percentage of the control absorbance at 570 nm [35].

#### 3.4.3. AlamarBlue^®^ Assay

Cells were plated into 96-well plates (3 × 10^5^ cells/mL). After 24 h, substances were added to each well using the HTS, and cells were incubated for 72 h. Control groups received DMSO. Twenty-four hours before the end of the incubation, 10 µL of a stock solution (0.436 mg/mL) of Alamar Blue (resazurin, Sigma-Aldrich Co., St. Louis, MO, USA) was added to each well. Absorbance was measured as above. The effect of each sample was quantified as the percentage of the control absorbance at 570 nm and 600 nm [36].

## 4. Conclusions

In the present study, two new 7-oxostaurosporine derivatives were obtained from the Brazilian endemic tunicate *Eudistoma vannamei*. The mixture of these compounds presented a strong antiproliferative effect against tumor cell lines, inducing a distinguished and persistent G2 arrest in sub-toxic concentrations, while at toxic concentrations, the treated cells underwent apoptosis. 

## Supplementary Files

Supplementary File 1: PDF-Document (PDF, 270 KB)
