# Supplementary material for: Structure Elucidation and Anticancer Activity of 7-Oxostaurosporine Derivatives from the Brazilian Endemic Tunicate Eudistoma vannamei
Source: Mar Drugs. 2012 May 21;10(5):1092–102. doi: 10.3390/md10051092 (PMC3397465; doi:10.3390/md10051092)

Supplementary Information

INDEX

Figure S1	Fractionation of the aqueous methanol extract of *Eudistoma vannamei*. The fractionation was guided by the cytotoxic activity against HL-60 leukemia cells using the MTT assay.	S2
Figure S2	^{1}H NMR spectrum (500 MHz, pyridine-d_5) of 2-hydroxy-7-oxostaurosporine (**1**) and 3-hydroxy-7-oxostaurosporine (**2**).	S3
Figure S3	^{13}C NMR spectrum (125 MHz, pyridine-d_5) of 2-hydroxy-7-oxostaurosporine (**1**) and 3-hydroxy-7-oxostaurosporine (**2**).	S4
Figure S4	2D HSQC spectrum (500 × 125 MHz, pyridine-*d*5) of 2-hydroxy-7-oxostaurosporine (**1**) and 3-hydroxy-7-oxostaurosporine (**2**).	S5
Figure S5	2D HMBC spectrum (500 × 125 MHz, pyridine-*d*5) of 2-hydroxy-7-oxostaurosporine (**1**) and 3-hydroxy-7-oxostaurosporine (**2**).	S6
Figure S6	2D COSY spectrum (500 × 500 MHz, pyridine-*d*5) of 2-hydroxy-7-oxostaurosporine (**1**) and 3-hydroxy-7-oxostaurosporine (**2**).	S7
Figure S7	ESI-HRMS spectrum of 2-hydroxy-7-oxostaurosporine (**1**) and 3-hydroxy-7-oxostaurosporine (**2**).	S8

Figure S1: Fractionation of the aqueous methanol extract of *Eudistoma vannamei*. The fractionation was guided by the cytotoxic activity against HL-60 leukemia cells using the MTT assay.

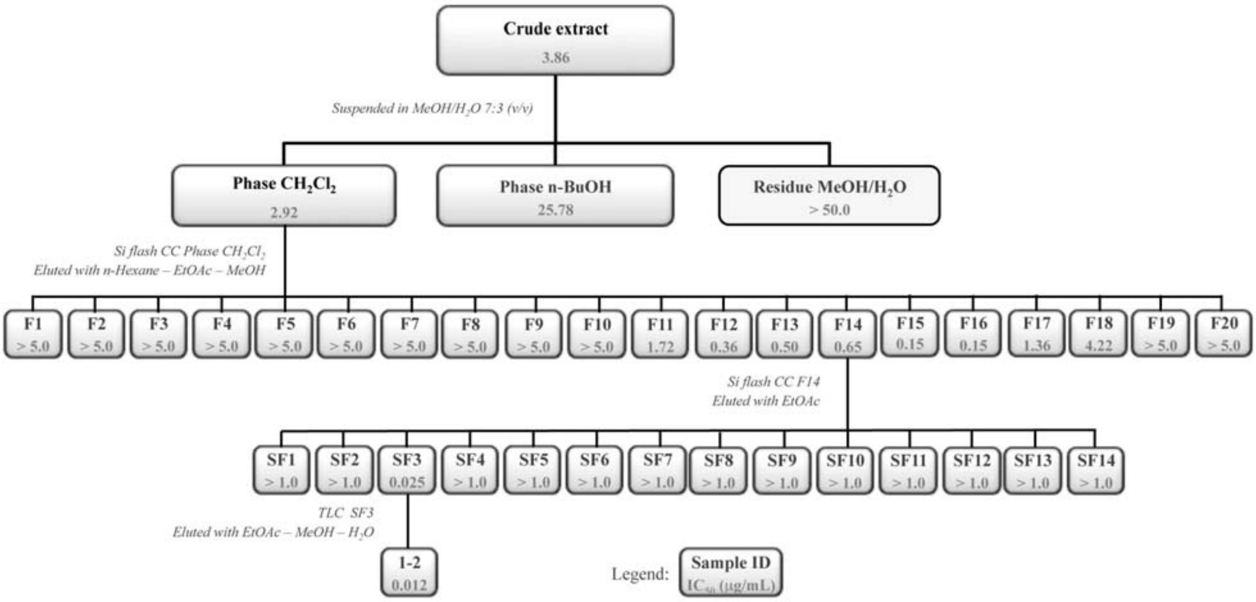

Figure S2: ^{1}H NMR spectrum (500 MHz, pyridine-d_5) of 2-hydroxy-7-oxostaurosporine (**1**) and 3-hydroxy-7-oxostaurosporine (**2**).

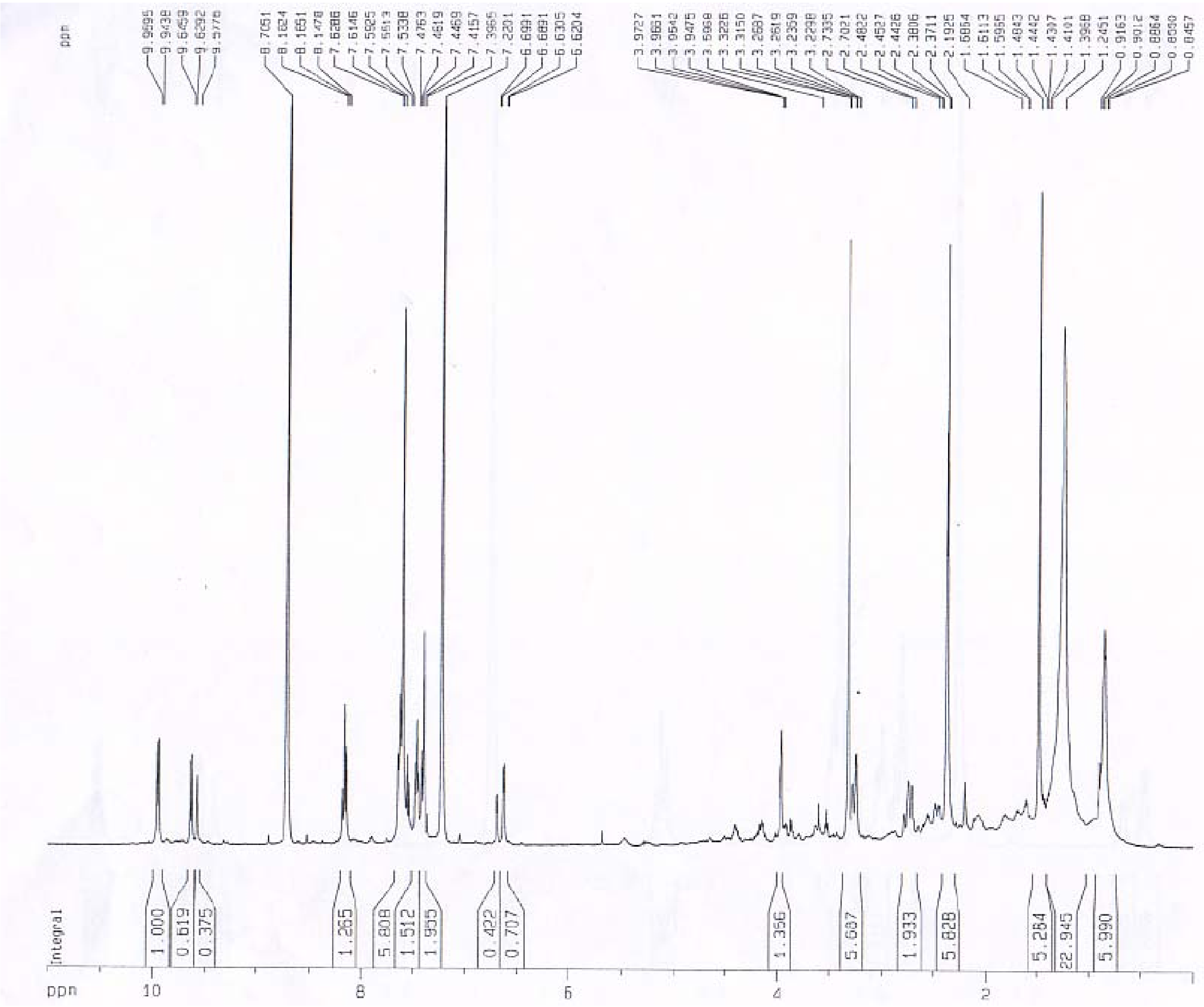

Figure S3: ^{13}C NMR spectrum (125 MHz, pyridine-d_5) of 2-hydroxy-7-oxostaurosporine (**1**) and 3-hydroxy-7-oxostaurosporine (**2**).

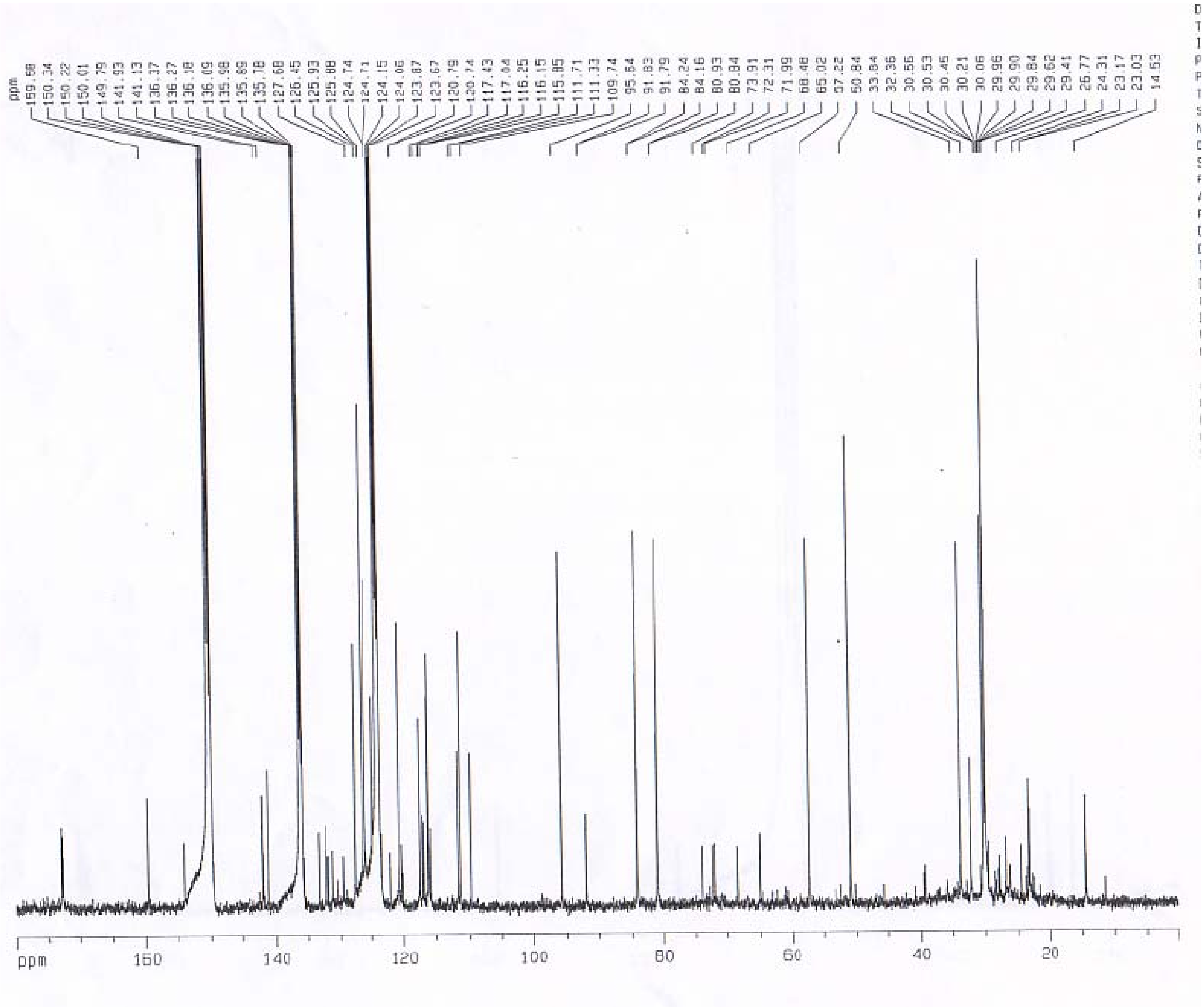

Figure S4: 2D HSQC spectrum (500 × 125 MHz, Pyr-d5) of 2-hydroxy-7-oxostaurosporine (**1**) and 3-hydroxy-7-oxostaurosporine (**2**).

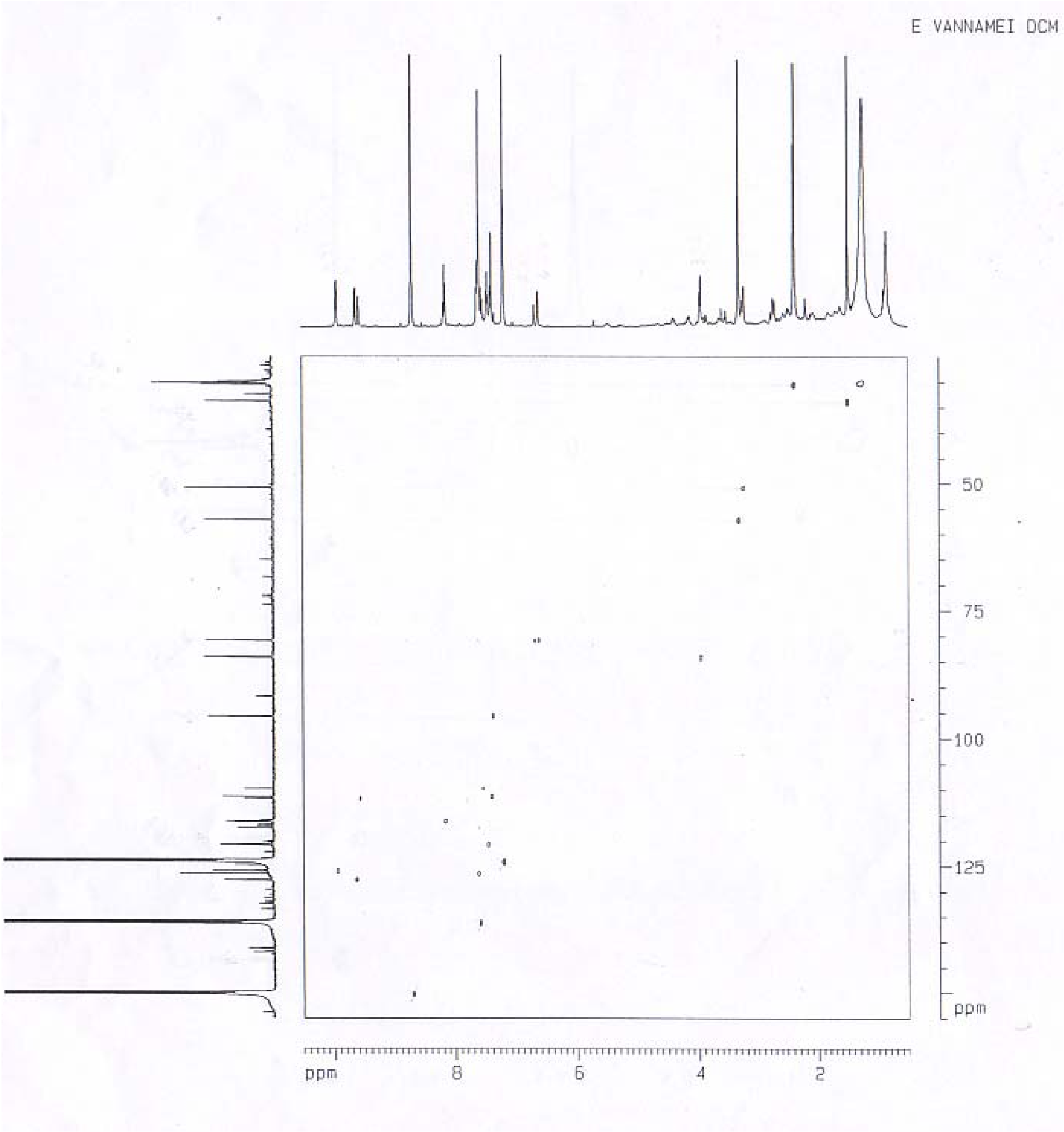

Figure S5: 2D HMBC spectrum (500 × 125 MHz, pyridine-*d*5) of 2-hydroxy-7-oxostaurosporine (**1**) and 3-hydroxy-7-oxostaurosporine (**2**).

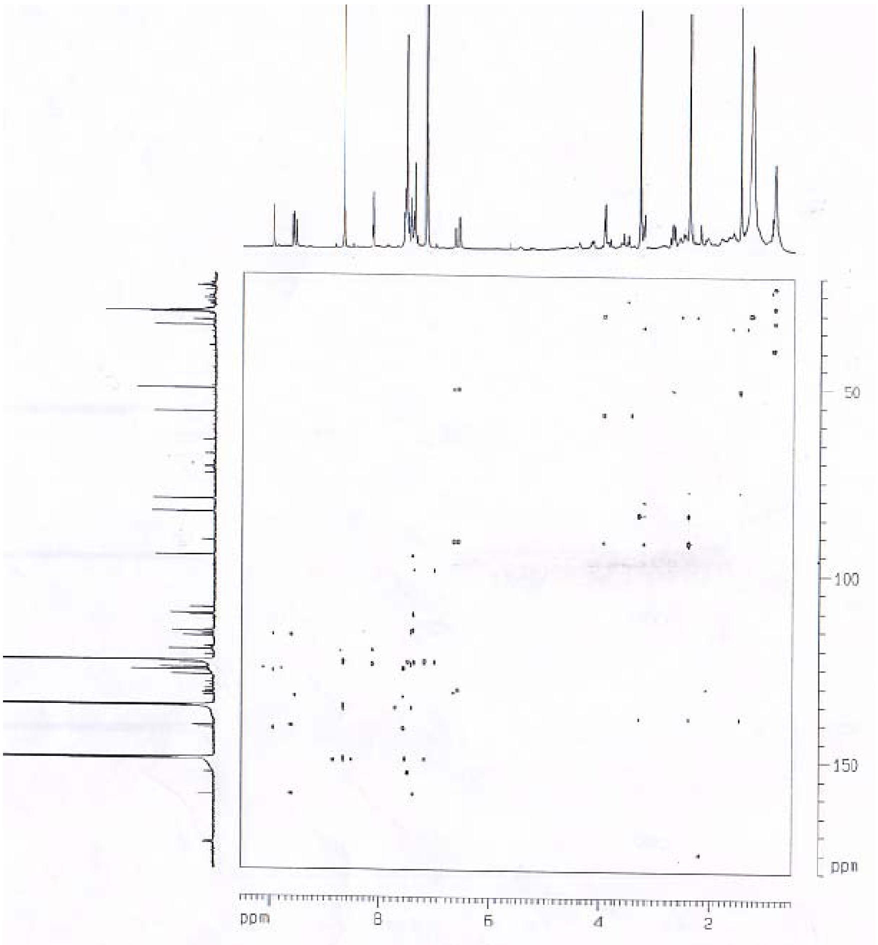

Figure S6: 2D COSY spectrum (500 × 500 MHz, pyridine-*d*5) of 2-hydroxy-7-oxostaurosporine (**1**) and 3-hydroxy-7-oxostaurosporine (**2**).

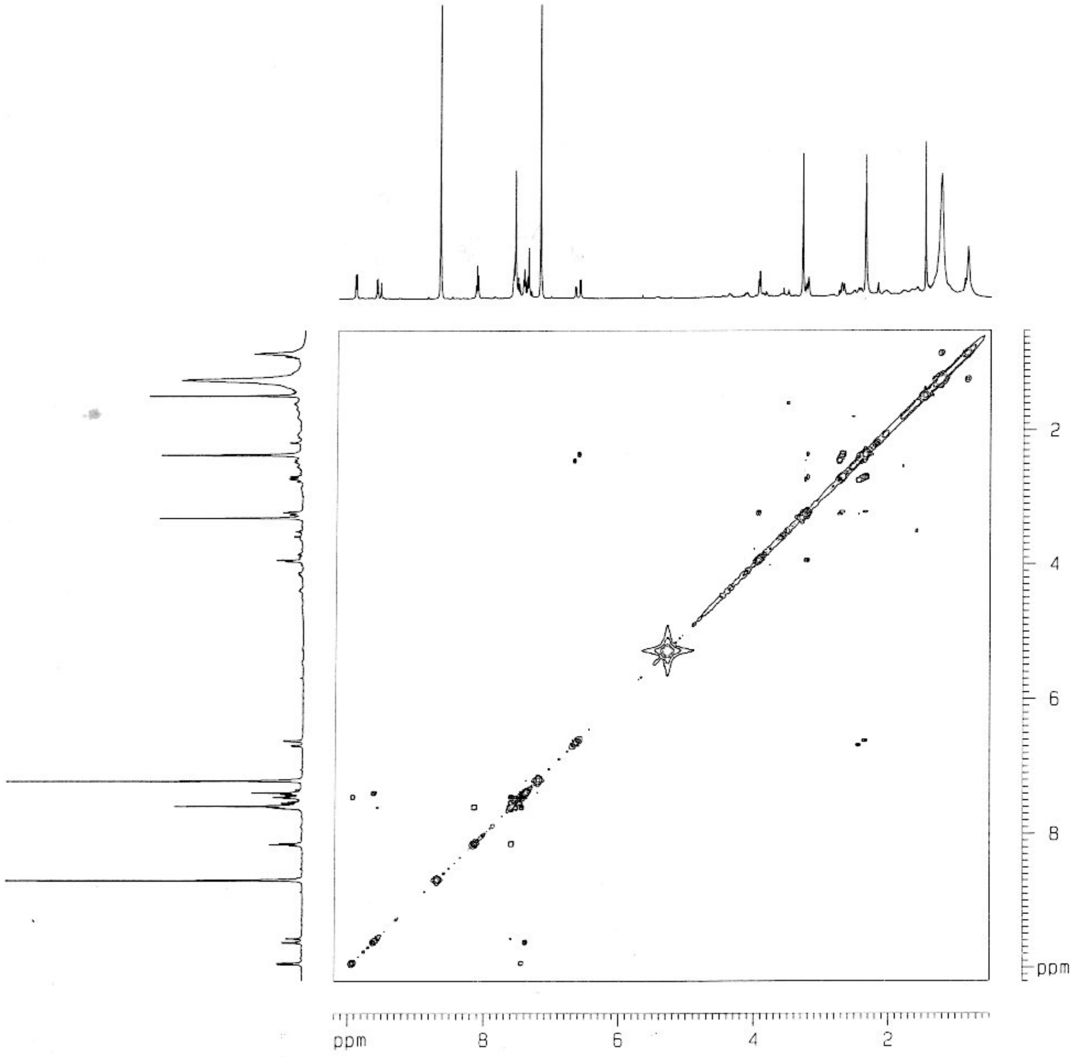

Figure S7: ESI-HRMS spectrum of 2-hydroxy-7-oxostaurosporine (**1**) and 3-hydroxy-7-oxostaurosporine (**2**).

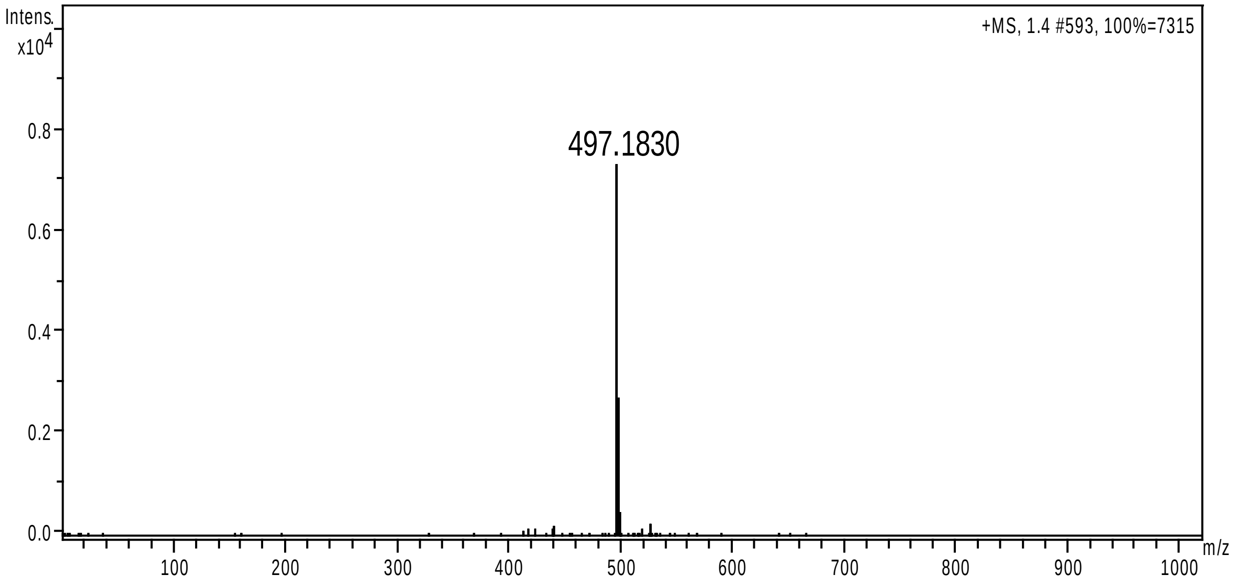

Supplement: Supplementary File 1: — PDF-Document (PDF, 270 KB) [file marinedrugs-10-01092-s001.pdf]
